# SCOPEOUT: sustainability and spread of quality improvement activities in long-term care- a mixed methods approach

**DOI:** 10.1186/s12913-018-2978-0

**Published:** 2018-03-12

**Authors:** Lisa A. Cranley, Matthias Hoben, Jasper Yeung, Carole A. Estabrooks, Peter G. Norton, Adrian Wagg

**Affiliations:** 1 Lawrence S. Bloomberg Faculty of Nursing, University of Toronto, 155 College Street, Toronto, ON Canada; 2 Faculty of Nursing, University of Alberta, 11405 87 Avenue NW, Edmonton, AB Canada; 3 Faculty of Medicine and Dentistry, Department of Medicine, University of Alberta, 11405 87 Avenue NW, Edmonton, AB Canada; 4 Department of Family Medicine, University of Calgary, 2500 University Drive NW, Calgary, AB Canada

**Keywords:** Sustainability, Spread, Quality improvement, Care aide, Residential long-term care facilities

## Abstract

**Background:**

Interventions to improve quality of care for residents of long-term care facilities, and to examine the sustainability and spread of such initiatives, remain a top research priority. The purpose of this exploratory study was to assess the extent to which activities initiated in a quality improvement (QI) collaborative study using care aide led teams were sustained or spread following cessation of the initial project and to identify factors that led to its success.

**Methods:**

This study used an exploratory mixed methods study design and was conducted in seven residential long-term care facilities in two Canadian provinces. Sustainability and spread of QI activities were assessed by a questionnaire over five time points for 18 months following the collaborative study with staff from both intervention with non-intervention units. Semi-structured interviews were conducted with care managers at six and 12 months. QI team success in applying the QI model was ranked as high, medium, or low using criteria developed by the research team. Descriptive statistics, bivariate analyses, and General Estimating Equations were used to analyze the data. Interview data were analyzed using thematic analysis.

**Results:**

In total, 683 surveys were received over the five time periods from 476 unique individuals on a facility unit. Seven managers were interviewed. A total of 533 surveys were analyzed. While both intervention and non-intervention units experienced a decline over time in all outcome measures, this decline was significantly less pronounced on intervention units. Facilities with medium and high success ranking had significantly higher scores in all four outcomes than facilities with a low success ranking. Care aides reported significantly less involvement of others in QI activities, less empowerment and less satisfaction with the quality of their work life than regulated care providers. Manager interviews provided evidence of sustainability of QI activities on the intervention units in four of the seven facilities up to 18 months following the intervention and demonstrated the need for continued staff and leadership engagement.

**Conclusion:**

Sustainability of a QI project which empowers and engages care aides is possible and achievable, but requires ongoing staff and leadership engagement.

**Electronic supplementary material:**

The online version of this article (10.1186/s12913-018-2978-0) contains supplementary material, which is available to authorized users.

## Background

The extent to which quality improvement (QI) interventions are sustained following the cessation of a formal QI project is important. Ideally, not only will the improvement activities be sustained, but there will also be spread of activities into areas where the original intervention was not implemented. Sustainability, defined as the maintenance of program activities beyond their initial funding period to continue achieving their desirable outcomes [1], and spread, the diffusion of the intervention components and processes beyond the initial area into which it is introduced [2], are always the goals of such interventions; however, evidence suggests that success in achieving these varies [3].

Improving care for cognitively impaired older adults residing in residential long-term care (LTC) facilities remain a top research priority [4]. LTC facilities perform like complex adaptive systems, characterized as nonlinear and unpredictable systems that are capable of undergoing spontaneous self-organization [5]. In accordance with complexity science, facilitating processes which encourage this self-organization, where humans adjust their interaction based on characteristics of the other parties and extensive communication can spread norms and create self-ordering structure, may increase the spread of effective practices across settings [2]. A microsystem, the clinical care unit, is itself a complex adaptive system that evolves over time [6]. A key feature to successful and sustainable microsystems (smaller units within an organization) is the retention and nurture of internal leaders and maintenance of a receptive and supportive organisational context [7]. However, relatively little is known about the processes required to sustain and spread QI activities within clinical microsystems and the larger organization [2, 8]. There is increasing recognition that the extent to which interventions are sustained is influenced by several factors, including characteristics of program design/intervention, organizational setting, the broader community environment [9], and processes and interactions [10], and a better understanding of these factors, beyond initial implementation efforts is needed [10].

The purpose of this study was to determine the extent to which activities initiated in a QI collaborative study were sustained or spread and to examine factors that led to the success of this QI initiative. The QI initiative Safer Care for Older Persons (in residential) Environments (SCOPE), used care aide (unregulated care providers) led QI teams [11, 12]. Our hypotheses based on the factors from literature were that (a) individual care provider characteristics (qualification, age, sex, experience), (b) facility characteristics (size, location, success as perceived by managers), and (c) having been exposed to the study intervention would influence sustainability and spread.

This study SCOPEOUT followed on from a 2 year pilot study (SCOPE) conducted in 10 units within seven residential LTC facilities in two Canadian Provinces: Alberta and British Columbia (BC) [11, 12]. This paper reports the findings of a follow-up study, SCOPEOUT, which addressed the following questions:

1. To what extent did staff QI teams continue to use QI processes and sustain improvements on SCOPE intervention units over an 18-month period?
2. To what extent did QI activities spread beyond the SCOPE intervention units?
3. What factors influenced the success of a QI initiative?
4. Was there a sustained impact on quality of work life or empowerment following the SCOPE intervention?

## Methods

### Study design

SCOPEOUT was an18-month mixed methods study that examined if and how improvements and activities achieved in the SCOPE pilot study were sustained and spread after the study ended. SCOPE focused on the front-line clinical unit (microsystem) where care is delivered [7]. The SCOPE study protocol and findings are reported elsewhere [11–13]. The mixed methods design included a longitudinal paper survey (five time points in 18 months, 2012–2013) and individual face-to-face semi-structured interviews.

### Setting and sample

The setting for this study was the seven LTC facilities that participated in the SCOPE study. In Canada, LTC is provided as public not-for-profit, voluntary not-for-profit (faith-based or private), or private for-profit [14, 15]. Staff eligible to complete the surveys were care aides, licensed practical nurses, registered nurses, allied healthcare providers and care managers who were able to identify a care unit in one of the seven facilities on which they had worked for at least 3 months. In addition, care aides needed to be able to identify a unit on which they worked over 50% of their time and to read and write in English. A quota sampling approach was used to invite all eligible staff to complete the survey. The target sample was 20 staff per facility for each time point. As this was an exploratory study, and there were no previous studies on effect sizes of outcomes assessed in this study (i.e., measures to assess sustainability and spread of the SCOPE intervention), we were unable to conduct an a priori sample size calculation. However, our results and effect sizes can inform sample size calculations for future studies of sustainability and spread of the SCOPE intervention or similar interventions.

Purposive sampling was used for the semi-structured interviews with the goal of including the seven unit care managers involved in the SCOPE project.

### Data collection

#### Surveys

A structured survey to determine the extent to which SCOPE activities were still in place on SCOPE intervention units and to what extent SCOPE activities had spread to non-intervention units was developed. The survey (Additional file 1) included participant demographic questions, questions on QI activities intended to improve resident care (e.g., use of Plan-Do-Study-Act [PDSA] cycles), inclusion of others into QI activities (e.g., by using shift reports), participants’ satisfaction with their quality of their work life, and staff empowerment (proxy measures to assess staff perceptions of their ability to be involved in or to enact change in their practice). An open text field in the survey was included where participants could write a narrative of any additional comments pertaining to QI activities, and two additional open text questions (included from Time 2 onwards) about staff understanding of and involvement in QI activities. Participants completed paper-based surveys, and were assigned a unique number so that the number of times the same staff member completed the survey (up to five times) could be determined.

#### Semi-structured interviews

Unit managers / care coordinators were interviewed twice during the study period to gain an understanding of QI activity in the facility at the time, the extent to which the QI work completed in the SCOPE study was sustained and/or spread beyond the intervention unit, and to gain an understanding of any other QI work introduced into the facility beyond the SCOPE study. Two members of the research team (JY, BW) conducted semi-structured face-to-face, audio-recorded interviews at six and 12 months following cessation of the SCOPE QI intervention (interview guide in Additional file 2). Interviews lasted 45–60 min.

### Data analysis

#### Surveys

Descriptive statistics, bivariate analyses (two-sided Fisher’s exact tests for categorical data and two-sided t-test for two independent samples for continuous data) and General Estimating Equations (GEEs) were used to analyze survey data. The number of missing items was small overall (below 5% for each variable, Additional file 3) and missing items were distributed completely at random. Therefore, any observation with missing items were deleted from the analysis [16].

The four outcome measures were: number of QI activities, number of ways to involve others in QI activities, empowerment, and satisfaction with quality of work life. With the exception of satisfaction with quality of work life (based on one item only), summary scores were created for the outcome measures. A summary score of QI activities reported by participants was generated by counting for each participant to how many of the nine activities (Additional file 1, questions 1–3) this person responded “yes.” A summary score for the seven ways to include others in QI activities (Additional file 1, question 8), using the same method was also generated. Finally, an empowerment score was created by averaging the agreement scores (1 = strongly disagree to 5 = strongly agree) of questions 4–7 (e.g., question 6: It is possible for me to make the changes to achieve our quality improvement goals on my unit) (Additional file 1).

For each individual survey item and for the summary scores, run charts to illustrate the proportion of yes-responses (for dichotomous items) or the average agreement score (for continuous outcomes) by study group (intervention versus non-intervention unit) and time of data collection were generated. For the three summary scores and the satisfaction with quality of work life item, additional run charts comparing scores by study group, time of data collection and team success in applying the SCOPE QI model (high, medium or low; criteria and methods for determining this success are described below) were created.

To assess sustainability and spread, GEEs for each individual survey item and for the summary scores as the dependent variable were run. The dependencies of multiple surveys collected from the same individual, and for clustering of responses collected within the same facility were accounted for. A log-link for dichotomous items, a maximum likelihood link for continuous outcomes, and an exchangeable covariance matrix in all models was used. The main independent variables in each model were (a) study group, (b) time of data collection and (c) an interaction effect between study group and time of data collection. Each model was adjusted for participant characteristics (age, sex, care aide versus regulated care staff, years of experience in current role and on care unit) and facility characteristics (province, size, owner operator model, success rank).

#### Semi-structured interviews

Manager interviews were transcribed verbatim and managed using the qualitative analysis software MAXQDA 11 (http://www.maxqda.com/) Thematic analyses [17], specifically focusing on topics related to sustainability and spread of the SCOPE intervention and factors positively or negatively affecting sustainability and spread was conducted. QI team success in applying the QI model was qualitatively ranked as high, medium or low based on six criteria developed by the research team: (1) QI team fidelity/adherence to the SCOPE intervention; (2) team engagement in QI activities; (3) manager support in QI activities; (4) QI team communication about SCOPE-related activities; (5) awareness or knowledge of the SCOPE study; and (6) staff continuation of the QI work (e.g., continued use of the PDSA model). Ranking was performed independently by three members of the research team using SCOPE study data that included research team field notes combined with measures addressing adherence to the QI process completed by the QI teams (work group cohesion, work group communication, inter-team relationships, and team progress towards improvement goal), and SCOPEOUT data (manager interviews). Final ranking of the facilities was agreed upon by research team consensus.

### Ethics approval

The study protocol was approved by the University of Alberta (Pro00012517_REN2), University of Calgary (ID: 23130), and the Interior Health Region of BC (ID 2010-022) research ethics boards. Operational approval from each facility and written informed consent from study participants prior to data collection was obtained.

## Results

### Sample description

A total of 533 surveys were included. Table 1 includes participant characteristics by time of data collection and Table 2 illustrates participant characteristics by study group. The largest proportion of participants (*n* = 268, 71.3%) completed the survey once, *n* = 68 (18.1%) completed the survey twice, *n* = 31 (8.2%) completed the survey three times, and *n* = 9 (2.4%) completed the survey four times. No one completed the survey five times.

**Table 1:** Participant characteristics by time of data collection

	Time 1	Time 2	Time 3	Time 4	Time 5
Number of surveys collected	114	99	119	95	106
Completed the survey first time, N (%)	–	62 (62.6%)	86 (72.3%)	57 (60.0%)	57 (53.8%)
Age range, N (%)					
< 25 years	3 (2.6%)	1 (1.0%)	3 (2.5%)	2 (2.1%)	1 (0.9%)
25-34 years	10 (8.7%)	7 (7.0%)	8 (6.7%)	5 (5.2%)	14 (13.1%)
35-44 years	35 (30.6%)	28 (28.2%)	36 (30.2%)	26 (27.3%)	25 (23.5%)
45-54 years	42 (36.7%)	44 (44.4%)	44 (36.9%)	47 (49.4%)	47 (44.2%)
> 54 years	23 (20.1%)	19 (19.1%)	27 (22.5%)	15 (15.7%)	19 (17.7%)
*Missing*	*1 (0.8%)*	*–*	*1 (0.8%)*	*–*	*–*
Sex, N (%)					
Male	16 (14.0%)	11 (11.1%)	5 (4.2%)	14 (14.7%)	15 (14.1%)
Female	98 (85.9%)	88 (88.8%)	114 (95.7%)	81 (85.2%)	90 (84.9%)
*Missing*	*–*	*–*	*–*	*–*	*1 (0.9%)*
Care provider group, N (%)					
Care aides	93 (81.5%)	81 (81.8%)	90 (75.6%)	83 (87.3%)	82 (77.3%)
Nurses	16 (14.0%)	12 (12.1%)	23 (19.3%)	10 (10.5%)	23 (21.6%)
Allied health providers	–	2 (2.0%)	1 (0.8%)	–	–
Managers	5 (4.3%)	4 (4.0%)	5 (4.2%)	2 (2.1%)	1 (0.9%)
Years worked in current role, M (SD)	12.95 (8.51)	13.36 (8.03)	12.38 (8.88)	12.02 (8.04)	13.18 (9.2)
Years worked on unit, M (SD)	7.04 (6.01)	7.9 (6.61)	7.74 (7.57)	6.27 (4.97)	8.3 (7.48)
Intervention unit, N (%)	45 (39.5%)	39 (39.4%)	42 (35.3%)	47 (49.5%)	42 (39.6%)

Note: N = number of individuals, % = percent of individuals, M = mean, SD = standard deviation

**Table 2:** Participant characteristics by study group

	Intervention units	Non-intervention units	*P*
Number of surveys collected	215	318	
Age range, N (%)			
< 25 years	5 (2.3%)	5 (1.6%)	< *0.0001*^a^
25-34 years	12 (5.6%)	32 (10.0%)	
35-44 years	50 (23.3%)	100 (31.4%)	
45-54 years	100 (46.5%)	124 (39.0%)	
> 54 years	47 (21.9%)	56 (17.6%)	
*Missing*	*1 (0.5%)*	*1 (0.3%)*	
Sex, N (%)			
Male	28 (13.0%)	33 (10.4%)	0.069^a^
Female	186 (86.5%)	285 (89.6%)	
*Missing*	1 (0.5%)	–	
Care provider group, N (%)			
Care aides	187 (87.0%)	242 (76.1%)	< *0.0001*^a^
Nurses	22 (10.2%)	62 (19.5%)	
Allied health providers	1 (0.5%)	2 (0.6%)	
Managers	5 (2.3%)	12 (3.8%)	
Years worked in current role, M (SD)	13.9 (9.18)	12.01 (8.03)	*0.015* ^b^
Years worked on unit, M (SD)	8.7 (6.97)	6.64 (6.32)	< *0.001*^b^

Note: N = number of individuals, % = percent of individuals, M = mean, SD = standard deviation

^a^Fisher’s exact test, two-sided (*p* < 0.05)

^b^T-test for two independent samples, two-sided (*p* < 0.05)

Additional file 3 lists descriptive statistics for each survey item by time of data collection.

The residential LTC facilities (Table 3) had a combined total of 34 units. Most had one designated SCOPE intervention unit although one of the large facilities had two (Facility A) and the other had three (Facility B). Two small facilities (Facility E and F) combined two to three units to form one intervention unit, making a total of 10 intervention units. There were 24 non-intervention units. The facilities were primarily publicly owned. Facilities were large in Alberta and most facilities in BC were small.

**Table 3:** Facility characteristics

Facility ID	Province	Facilitysize^a^	Ownership type	Numberof units	Clinicalarea	Successranking^b^
A	Alberta	Large	Public	6	Pain, behaviour	Low
B	Alberta	Large	Voluntary	8	Pain, skin care, behaviour	Medium
C	British Columbia	Medium	Public	2	Skin care	Medium
D	British Columbia	Small	Public	4	Pain	Low
E	British Columbia	Small	Public	6	Behaviour	High
F	British Columbia	Small	Public	6	Behaviour	High
G	British Columbia	Small	Public	2	Pain	Medium

^a^Small: < 100 beds; Medium 100–200 beds; Large range > 200 beds

^b^Qualitative ranking of each facility’s success in applying the SCOPE quality improvement model

### QI activities (intervention vs non-intervention units)

**Fig. 1:**
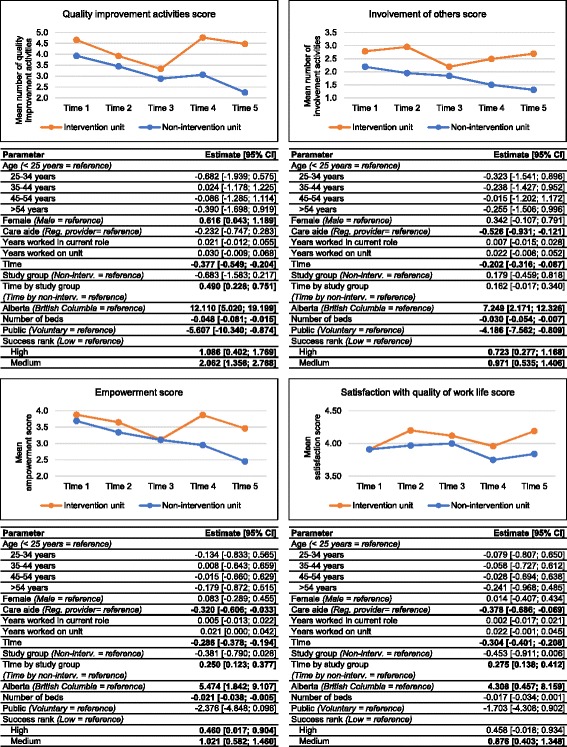
General Estimating Equation results of the SCOPEOUT survey outcome scores. Legend: 95% CI = 95% confidence interval

Figure 1 includes run charts and GEE results of the SCOPEOUT survey outcome scores. While both intervention and non-intervention units experienced a decline over time in all outcome measures, this decline was statistically significantly less pronounced on intervention units. Overall, the study group assignment did not independently predict differences in the four scores. However, the interaction term (time by study group) was independently associated with three of the four outcome scores. With each additional point in time, the average number of QI activities on intervention units increased by an additional 0.5 points over the average number of QI activities on non-intervention units. Empowerment and satisfaction with quality of work life scores also decreased more slowly over time on intervention units than on non-intervention units. Facilities with medium and high success ranking had significantly higher scores in all four outcomes than facilities with a low success ranking. Age, experience on unit and experience in current role were not significantly associated with the outcome scores. However, females reported significantly more QI activities than males. Care aides reported significantly less involvement of others in QI activities, less empowerment and less satisfaction with their work life than regulated care providers. Scores for all four outcomes were higher in facilities located in Alberta, compared to facilities in BC. Additionally, the more beds a facility had the lower were the: number of QI activities, number of ways to involve others in QI activities, and empowerment scores. Public facilities had lower QI and involvement scores than voluntary facilities.

All findings were confirmed by the GEE results of the individual survey items (Additional file 4), providing evidence for sustainability of the SCOPE intervention on intervention units. The QI activity most specific to the SCOPE study was the use of SCOPE binders, which comprised information provided by the study team (including clinical experts), such as QI tools and research evidence to support best practices for each of the three clinical care areas for improvement (i.e., dementia related behaviour management, skin care/pressure ulcer management, and pain management) [12]. Our finding that 28%–45% of the non-intervention unit participants reported use of the SCOPE binders (Additional file 4) provided evidence for spread of SCOPE activities to non-intervention units.

### Interview data

#### Sustainability and spread

Managers’/ care coordinators’ perceptions of the level of SCOPE sustainability and spread were largely consistent over time (at six and 12 months). Findings from the interviews indicated sustainability of QI activities on the intervention units in four of the seven facilities up to 18 months after the SCOPE intervention ended (Table 4).

**Table 4:** Level of SCOPE sustainability and spread based on manager interview data

SCOPEOUT facility rankings^a^	Sustained	Sustained and embedded	Spread
1. Facility F	√	√	√
2. Facility E	√		√
3. Facility C	√		√
4. Facility B			
5. Facility G	√	√	
6. Facility D			
7. Facility A			

^a^Facility from highest ranked to lowest ranked

In Facility F, the highest ranked facility in the SCOPEOUT study (Table 4), the manager indicated that their team not only sustained their work from the SCOPE study, but were renamed from the SCOPE team to the QI team and continued to apply their knowledge of QI methods (e.g., PDSA) to other QI projects. The manager described how the QI team continued to work according to the improvement model and principles learned in SCOPE and it became embedded in their practice as “*a way of life for us*.” Though QI teams had not been formally established on other units in Facility F, the manager observed some indication of spread in that staff from units not involved in the SCOPE study approached the SCOPE team with problems to be solved; these non-SCOPE staff members became known as “*associate members*” of the QI team.

In Facility E, the second highest ranked facility in SCOPEOUT, the manager indicated that changes implemented as a result of the SCOPE intervention to improve dementia-related behaviours were sustained, and the original SCOPE team remained intact, visible and continued to use PDSA cycles for other QI work. Similar to Facility F, the team invited non-SCOPE staff members to provide feedback and input into ongoing work. Although spread of QI activities to other units did not occur (the manager suggested that this may be because the other units were in another building), the manager indicated that meetings were held to raise awareness among all staff about SCOPE and their QI team. In Facility C, the third highest ranked facility in SCOPEOUT, the manager described how staff continued to use the practices and measurement tools developed by the (still existing) SCOPE team in both the intervention unit and non-intervention units. The use of this tool also spread to two other facilities participating in the SCOPE intervention, at the request of staff in those facilities. There was also evidence of sustainability occurring in Facility G (a lower ranked facility). Though the SCOPE team no longer existed, staff applied the QI principles and skills learned in the SCOPE project to other work including a falls program and accreditation, to the point where the manager described it as routine practice. The manager acknowledged that SCOPE empowered the care aides and made them realize that “*they have a voice*.” The manager also noted that the SCOPE project improved teamwork, communication, and problem-solving skills.

Based on the interview data, for three of the seven facilities (Facility A, B, D) the SCOPE intervention was neither sustained nor spread, despite one team’s efforts in Facility B to sustain the work. The manager indicated that the reason for this was the lack of resources (financial, human) necessary to continue. Facility A underwent several organizational and operational changes (e.g., changes in leadership) and Facility D was unable to sustain the work due to competing demands and priorities from the government. Managers described several other factors that affected success and potential sustainability of SCOPE such as, turnover of key QI team members, staff resistance to change, and time constraints. However, managers also described factors that fostered success of SCOPE, such as facilitating team dynamics, removing barriers, doing the SCOPE work properly and in small steps, having commitment from the QI team to do the work and seek buy- in from other staff, and the improvements were observable. Managers described employing several leadership strategies to engage and support staff during SCOPE such as, intervening only when needed, emphasizing their own commitment to SCOPE, and providing praise and recognition for the QI team’s efforts.

## Discussion

### QI activities (intervention vs non-intervention units)

Sustainability and spread are difficult to achieve and require collaboration, engagement and partnership on the part of those involved [18]. Organizational size, owner operator model, and province each contributed independently to the scores. Overall, larger nursing facilities, on average, had lower scores (e.g., less reported QI activities) than smaller ones, and public facilities scored lower than the voluntary facility. In a previous study, our team found that differences in LTC facility owner operator model and province can significantly influence best practice use in residential LTC settings [19]. Facility size is also a factor that can influence quality of care and resident outcomes [20]. Findings from a recent systematic review found that smaller LTC facilities (measured by number of beds) were more likely to deliver higher quality and better outcomes for residents than larger facilities [20]. However, larger facilities may have a greater likelihood of early innovation adoption than smaller facilities [21]. In a systematic review that compared quality of care in for-profit and not-for profit (publicly and privately owned) LTC facilities, most studies suggested a trend towards higher quality care in not-for-profit facilities than in for-profit facilities, while acknowledging that many other factors would likely influence this relationship [22]. A recent study supports this finding [23]; however, what causes these differences warrants further research.

While this study showed a decreasing trend in continued QI activity on intervention units over time, there was evidence of spread of SCOPE activities (use of SCOPE binders) to non-intervention units. Presence of the binders on intervention units may have spread to other staff working on non-intervention units through word of mouth. At Time 4, there was a significant increase in outcome scores (with the exception of satisfaction with quality of work life) for intervention units. This may be due to bias in responses from learning effects of those participants who completed the survey more than once (*n* = 108) or from factors outside the intervention. We also found that the sex of workers influenced the number of reported QI activities in the unit, which may warrant further research and analysis by sex.

Interview data indicated sustainability of QI activities in intervention units in four of the seven facilities (including the top three ranked facilities F, E, C) 18 months after the SCOPE intervention was implemented, and in two of these four facilities, SCOPE QI activities were embedded into routine practice (Facility F and G). Managers/care coordinators from the top three ranked facilities described how the SCOPE intervention spread within the SCOPE intervention unit or beyond the facility.

### Staff empowerment and quality of work life

Higher ranked facilities had higher empowerment and satisfaction with quality of work life scores than facilities with a low success ranking. However, care aides reported less empowerment and satisfaction with quality of work life than regulated staff. Care aides have been described as an invisible and marginalized workforce [24] - they do not always have access to the resident care plan, attend resident care conferences, or feel that their concerns are addressed by the team [25]. A recent systematic review found that individual factors influencing care aides’ job satisfaction were empowerment and autonomy, while organizational factors included facility resources and workload [26]. Cready et al. reported survey findings from care aides and nurses from facilities where care aide empowered teams had been implemented and from facilities with more traditional management approaches, and found that feelings of high empowerment among care aides were associated with higher assessments of their job performance and job satisfaction [27]. Indeed, strategies to empower the care aide workforce may draw on their high levels of job efficacy, that is, a sense of their work’s worth [12, 28].

There was concern from the research team regarding the creation of expectations which might be unfulfillable on the part of empowered and trained care aides following the SCOPE study. However, there was some evidence that this was not the case; after the SCOPEOUT study, three care aides who led their SCOPE QI teams and the two regional decision-makers from the SCOPE study were interviewed. Both the care aides and decision-makers reported no unintended consequences as a result from participating in the SCOPE project, and in fact, they described how they had learned and benefitted from the experience.

### Factors that influenced success of the SCOPE pilot study

Those facilities that had greater success in sustaining the SCOPE work were able to apply the QI methods and skills learned to other projects and engage other staff by eliciting their feedback and input. For two facilities, strong management leadership facilitated the QI process to become embedded into routine practices to improve resident care. Managers interviewed described improvements in staff teamwork, communication and problem-solving abilities as factors leading to successful continuation. While these individual and unit-level (microsystem) factors influenced success of the QI initiative, it was primarily organizational / system level factors (e.g., leadership change, competing priorities, lack of resources) that influenced the extent of sustainability or spread of the intervention.

Literature reviews have identified a broad range of factors influencing program/intervention sustainability, including organizational context (e.g., leadership, culture), capacity (e.g., resources, champions, staffing), stakeholder support, intervention characteristics (e.g., adaptability) and implementation processes (e.g., evaluation, feedback) [10, 29, 30]. In this study, organizational factors played a key role in sustainability of QI activities, where strong leadership and staff engagement were the main factors. Findings from this study are consistent with those from the high- and low-performing organizational literature; high-performing organizations have strong leadership support, good communication, and teamwork [8, 31–33]. Valuing the importance of staff and fostering staff appreciation are key leadership behaviours in high-performing LTC facilities [31, 33]. Other high-performance work practices include a flattened supervisory structure, increased autonomy for frontline workers, and self-managed teams [33, 34]. Such work practices associated with frontline health care worker high job satisfaction and perceived quality of care involves a combination of supervisor support, performance-based incentives, team-based work, and flexible work [35]. Successful teams in SCOPE, confirmed by SCOPEOUT, described the need to include persistent champions for the work to succeed, including ongoing staff and leadership commitment and engagement. The clinical microsystem literature highlights the growing importance of engaging staff and motivating them to develop and use their full potential [36, 37]. By optimizing the work environment, the clinical microsystem can achieve high levels of performance [36].

### Strengths and limitations of the study

This study investigated the rate at which SCOPE QI activities were sustained and spread, following the cessation of the initial intervention and external support. However, only one quarter of our sample completed the survey up to four times, limiting the ability to make a longitudinal comparison. SCOPE related activities were not well differentiated by staff from other QI initiatives which were being promoted by the region at subsequent times; this may be because there were other QI activities occurring at the same time at some facilities (e.g., hydration monitoring, bathing initiative). QI, as understood and expressed by the researcher, was not a well understood concept by staff respondents, who often interpreted this as activities to improve resident quality of life. This difference in interpretation of the meaning of the survey items might have led to inaccurate responses.

Our models (GEEs) account for the complexity and multiple dependencies of repeated assessments nested with participants and participants nested within care units and facilities. The number and rate of missing items was small and responses were missing at random – an important assumption of GEEs. GEEs are fairly robust against non-normal distributions, reinforcing our confidence in our models. Multiple significant effects indicate sufficient power for these complex models.

Although the LTC facility rankings were informed by data, they were subjectively derived based on research team consensus, and this may have limited the conclusions drawn. There was also a possibility of research “fatigue” even when answering quarterly surveys, as 28.7% of respondents completed the survey at least more than once. The field staff noted that some respondents appeared to give answers that they might have thought the researcher wanted to hear when repeatedly asked.

### Practice implications

The ability of a collaborative to create a sustaining infrastructure is seldom considered in published reports. This study has shed light on the natural history of sustainability of a QI project following the cessation of the initial intervention and support. Data have confirmed the multifactorial nature of the effort required to sustain an innovation and the potentially major effect of leader support on performance. This study demonstrates the implications for leadership and the potential of empowered staff in sustaining QI processes and improving quality of care delivery. There remains, however, a need for continuous use of valid data in a usable form at the level of the frontline staff delivering care in order for this potential to be unlocked and developed [38]. Strategies to ensure support for managers engaged in QI initiatives in LTC facilities including opportunities for leadership coaching are also needed. Such plans should also support staff involved in these initiatives and include education and coaching, the provision of dedicated time for QI activities, and ensure that small wins are acknowledged or celebrated to increase visibility of the work and to foster an ongoing commitment to sustaining improvements.

## Conclusion

Sustainability of a QI project which empowers and engages care aides is possible and achievable. Such sustainability requires ongoing staff and leadership engagement. Further research is needed into the factors that may facilitate scale up and spread beyond the initial microsystem into which the intervention was introduced.

## Additional files

Additional file 1: SCOPEOUT survey. (PDF 245 kb)

Additional file 2: SCOPEOUT interview guide. (PDF 203 kb)

Additional file 3: Descriptive statistics for each SCOPEOUT survey item by time of data collection. (PDF 165 kb)

Additional file 4: General Estimating Equation results of the SCOPEOUT survey items. (PDF 853 kb)

## Abbreviations

BC: British Columbia
GEE: Generalized estimating equation
LTC: Long-term care
PDSA: Plan-Do-Study-Act
QI: Quality improvement
SCOPE: Safer Care for Older People (in residential) Environments
