# Supplementary material for: SCOPEOUT: sustainability and spread of quality improvement activities in long-term care- a mixed methods approach
Source: BMC Health Serv Res. 2018 Mar 12;18:174. doi: 10.1186/s12913-018-2978-0 (PMC5848563; doi:10.1186/s12913-018-2978-0)
Supplement: Supplementary file 1 — SCOPEOUT survey. (PDF 245 kb) [file 12913_2018_2978_MOESM1_ESM.pdf]

SCOPE Follow-Up Quality Improvement Questionnaire

Data Collection Period:
Date:
Facility name:
Unit name:
Participant ID Number:

About you:

1. Please tell us your primary role on the unit where you work for most of the time?

   □1 HCA (Health Care Aide)
   □2 PCA (Personal Care Aide / Attendant)
   □3 RCA (Resident Care Aide)
   □4 Nursing Attendant
   □5 LPN (Licensed Practical Nurse)
   □6 RN (Registered Nurse)
   □7 PT (Physical/Physiotherapist)
   □8 OT (Occupational Therapist)
   □9 Other: (Please specify)

2. How long have your worked in this role?

   years months

3. How long have you worked on the unit where you work for most of the time?

   years months

4. Are you

   □1 male or □2 female?

5. Please indicate your age group (in years):

☐1 <20
☐2 20-24
☐3 25-29
☐4 30-34
☐5 35-39
☐6 40-44
☐7 45-49
☐8 50-54
☐9 55-59
☐10 60-64
☐11 65-70
☐12 >70

Quality improvement (QI) activities

The following questions refer to quality improvement activity on your unit meant that are intended to improve the quality of care for residents.. You may or may not be involved in these activities.

1. Are there currently any quality improvement activities on the unit?
   ☐1 Yes if Yes, proceed to the next question
   ☐2 No ***if No, skip to Question 9***

2. Does your quality improvement activity involve Plan- Do –Study- Act (PDSA) cycles?
   ☐1 Yes
   ☐2 No

3. Does your quality improvement team use any of the following information about your quality improvement activity at your meetings? **(CHECK ALL THAT APPLY)**
   ☐1 Information from SCOPE binders
   ☐2 Information on current activity with residents
   ☐3 Your local facility resident assessment indicators
   ☐4 New published research evidence in your quality area
   ☐5 Discussing resident care plans on your quality improvement area
   ☐6 Discussing new developments in care for your residents
   ☐7 Other, please specify:

4. To what extent do you agree or disagree with the following statement: I am involved in the QI activities in my unit based team?

Strongly Disagree	Disagree	Neither Agree Nor Disagree	Agree	Strongly Agree
☐1	☐2	☐3	☐4	☐5

5. To what extent do you agree or disagree with the statement:
The person or persons to whom I report most of the time is involved in my QI team's activity?

Strongly Disagree	Disagree	Neither Agree Nor Disagree	Agree	Strongly Agree
☐1	☐2	☐3	☐4	☐5

6. To what extent do you agree or disagree with the statement:
It is possible for me to make the changes to achieve our quality improvement goals on my unit?

Strongly Disagree	Disagree	Neither Agree Nor Disagree	Agree	Strongly Agree
☐1	☐2	☐3	☐4	☐5

7. To what extent do you disagree or agree with the following statement: I can make the changes needed on my unit to improve resident care?

Strongly Disagree	Disagree	Neither Agree Nor Disagree	Agree	Strongly Agree
☐1	☐2	☐3	☐4	☐5

8. In what ways do you or your team include others on the unit in your work? **(CHECK ALL THAT APPLY)**
   - ☐1 Shift reports
   - ☐2 Storyboards
   - ☐3 Whiteboard
   - ☐4 Storytelling
   - ☐5 Role modeling
   - ☐6 Weekly group huddles
   - ☐7 Other, please specify:

If you have answered questions 2 – 8, there is no need to answer questions 9 and 10.

9. Why do you think there are no quality improvement activities on your unit?
   - ☐1 Not enough time
   - ☐2 No need for QI on the unit
   - ☐3 No interest
   - ☐4 Not part of my or our role
   - ☐5 Insufficient support
   - ☐6 Other, please specify:

10. When were you last involved in or aware of any such activity? (Please tell us the last month and year that you recall a project on your unit)

    Month Year

<u>Quality of your working life</u>

To what extent do you agree or disagree with the following statement?
Overall I am satisfied with the quality of my work life.

Strongly Disagree	Disagree	Neither Agree Nor Disagree	Agree	Strongly Agree
☐1	☐2	☐3	☐4	☐5

<u>Additional Comments:</u>

SCOPE Follow-Up Quality Improvement Questionnaire

Quality improvement (QI) activities – additional questions

Note: the following two questions are added from Time 2 data collection

1. In what Quality Improvement activities are **you** involved?

2. Please tell us exactly what Quality Improvement activities **you** do?
