# Supplementary material for: SCOPEOUT: sustainability and spread of quality improvement activities in long-term care- a mixed methods approach
Source: BMC Health Serv Res. 2018 Mar 12;18:174. doi: 10.1186/s12913-018-2978-0 (PMC5848563; doi:10.1186/s12913-018-2978-0)
Supplement: Supplementary file 2 — SCOPEOUT interview guide. (PDF 203 kb) [file 12913_2018_2978_MOESM2_ESM.pdf]

Interview question guide for facility managers/care coordinators

Current QI activities:

1. What types of quality improvement activities are taking place in your facility?
2. Are they related to Scope in any way?
3. Are any of these QI activities led by Health Care Aides? Why or why not?
4. Are there any QI initiatives from policy or decision makers in your health region? (eg. Provincially/government-related)

SCOPE :

1. What was your impression of SCOPE? Did it benefit your facility in any way?
2. What is the current status of the SCOPE team? What types of QI activities are occurring within the SCOPE team?
3. How were you involved in SCOPE?
4. Are there any SCOPE tools or techniques that you continue to use?
5. What are some constraints to adopting a QI program such as SCOPE?
6. What does your facility do in regards to providing feedback and results from QI programs for your staff?
7. What does your facility do in terms of providing research evidence and best practise to the staff?
8. Are there any major changes in the overall staff structure the past 6 months? past year?
9. Did any other events take place recently that may deter the successful implementation and adoption of QI programs?
