# Supplementary material for: SCOPEOUT: sustainability and spread of quality improvement activities in long-term care- a mixed methods approach
Source: BMC Health Serv Res. 2018 Mar 12;18:174. doi: 10.1186/s12913-018-2978-0 (PMC5848563; doi:10.1186/s12913-018-2978-0)
Supplement: Supplementary file 3 — Descriptive statistics for each SCOPEOUT survey item by time of data collection. (PDF 165 kb) [file 12913_2018_2978_MOESM3_ESM.pdf]

Additional File 3: Survey items by time of data collection, including rates of missing items

		Time 1	Time 2	Time 3	Time 4	Time 5
**Quality improvement activities**, N (%)						
Are there currently any quality improvement activities on the unit?	Yes	108 (94.7%)	84 (84.8%)	85 (71.4%)	80 (84.2%)	67 (63.2%)
	No	6 (5.3%)	15 (15.2%)	34 (28.6%)	15 (15.8%)	39 (36.8%)
Does your quality improvement activity involve Plan-Do-Study-Act (PDSA) cycles?	Yes	92 (80.7%)	70 (70.7%)	69 (58.0%)	56 (58.9%)	56 (52.8%)
	No	11 (9.6%)	10 (10.1%)	14 (11.8%)	22 (23.2%)	11 (10.4%)
	NA	6 (5.3%)	15 (15.2%)	34 (28.6%)	15 (15.8%)	39 (36.8%)
	Missing	5 (4.4%)	4 (4.0%)	2 (1.7%)	2 (2.1%)	—
Does your quality improvement team use information from SCOPE binders at your meetings?	Yes	58 (50.9%)	46 (46.5%)	40 (33.6%)	45 (47.4%)	44 (41.5%)
	No	47 (41.2%)	37 (37.4%)	45 (37.8%)	35 (36.8%)	23 (21.7%)
	NA	6 (5.3%)	15 (15.2%)	34 (28.6%)	15 (15.8%)	39 (36.8%)
	Missing	3 (2.6%)	1 (1.0%)	—	—	—
Does your quality improvement team use information on current activity with residents at your meetings?	Yes	76 (66.7%)	56 (56.6%)	61 (51.3%)	61 (64.2%)	58 (54.7%)
	No	29 (25.4%)	27 (27.3%)	24 (20.2%)	19 (20.0%)	9 (8.5%)
	NA	6 (5.3%)	15 (15.2%)	34 (28.6%)	15 (15.8%)	39 (36.8%)
	Missing	3 (2.6%)	1 (1.0%)	—	—	—
Does your quality improvement team use your local facility resident assessment indicators at your meetings?	Yes	52 (45.6%)	42 (42.4%)	40 (33.6%)	44 (46.3%)	39 (36.8%)
	No	53 (46.5%)	41 (41.4%)	45 (37.8%)	36 (37.9%)	28 (26.4%)
	NA	6 (5.3%)	15 (15.2%)	34 (28.6%)	15 (15.8%)	39 (36.8%)
	Missing	3 (2.6%)	1 (1.0%)	—	—	—
Does your quality improvement team use new published research evidence in your quality area at your meetings?	Yes	27 (23.7%)	17 (17.2%)	20 (16.8%)	26 (27.4%)	20 (18.9%)
	No	78 (68.4%)	66 (66.7%)	65 (54.6%)	54 (56.8%)	47 (44.3%)
	NA	6 (5.3%)	15 (15.2%)	34 (28.6%)	15 (15.8%)	39 (36.8%)
	Missing	3 (2.6%)	1 (1.0%)	—	—	—
Does your quality improvement team use discussing resident care plans on your quality improvement area at your meetings?	Yes	85 (74.6%)	59 (59.6%)	61 (51.3%)	67 (70.5%)	58 (54.7%)
	No	20 (17.5%)	24 (24.2%)	24 (20.2%)	13 (13.7%)	9 (8.5%)
	NA	6 (5.3%)	15 (15.2%)	34 (28.6%)	15 (15.8%)	39 (36.8%)
	Missing	3 (2.6%)	1 (1.0%)	—	—	—
Does your quality improvement team use discussing new developments in care for your residents at your meetings?	Yes	76 (66.7%)	56 (56.6%)	62 (52.1%)	67 (70.5%)	53 (50.0%)
	No	29 (25.4%)	27 (27.3%)	23 (19.3%)	13 (13.7%)	14 (13.2%)
	NA	6 (5.3%)	15 (15.2%)	34 (28.6%)	15 (15.8%)	39 (36.8%)
	Missing	3 (2.6%)	1 (1.0%)	—	—	—
Does your quality improvement team use other information about your quality improvement activity at your meetings?	Yes	6 (5.3%)	14 (14.1%)	9 (7.6%)	5 (5.3%)	4 (3.8%)
	No	98 (86%)	69 (69.7%)	76 (63.9%)	75 (78.9%)	63 (59.4%)
	NA	6 (5.3%)	15 (15.2%)	34 (28.6%)	15 (15.8%)	39 (36.8%)
	Missing	4 (3.5%)	1 (1.0%)	—	—	—
**Inclusion of others**, N (%)						
Do you or your team include others on the unit in your work by shift reports?	Yes	105 (92.1%)	80 (80.8%)	82 (68.9%)	78 (82.1%)	62 (58.5%)
	No	3 (2.6%)	4 (4.0%)	3 (2.5%)	2 (2.1%)	5 (4.7%)
	NA	6 (5.3%)	15 (15.2%)	34 (28.6%)	15 (15.8%)	39 (36.8%)
Do you or your team include others on the unit in your work by storyboards?	Yes	19 (16.7%)	22 (22.2%)	22 (18.5%)	13 (13.7%)	19 (17.9%)
	No	89 (78.1%)	62 (62.6%)	63 (52.9%)	67 (70.5%)	48 (45.3%)
	NA	6 (5.3%)	15 (15.2%)	34 (28.6%)	15 (15.8%)	39 (36.8%)
Do you or your team include others on the unit in your work by whiteboard?	Yes	48 (42.1%)	44 (44.4%)	43 (36.1%)	31 (32.6%)	40 (37.7%)
	No	60 (52.6%)	40 (40.4%)	42 (35.3%)	49 (51.6%)	27 (25.5%)
	NA	6 (5.3%)	15 (15.2%)	34 (28.6%)	15 (15.8%)	39 (36.8%)
	Yes	18 (15.8%)	22 (22.2%)	20 (16.8%)	14 (14.7%)	20 (18.9%)

		Time 1	Time 2	Time 3	Time 4	Time 5
Do you or your team include others on the unit in your work by storytelling?	No	90 (78.9%)	62 (62.6%)	65 (54.6%)	66 (69.5%)	47 (44.3%)
	NA	6 (5.3%)	15 (15.2%)	34 (28.6%)	15 (15.8%)	39 (36.8%)
Do you or your team include others on the unit in your work by role modeling?	Yes	34 (29.8%)	24 (24.2%)	27 (22.7%)	23 (24.2%)	23 (21.7%)
	No	74 (64.9%)	60 (60.6%)	58 (48.7%)	57 (60.0%)	44 (41.5%)
	NA	6 (5.3%)	15 (15.2%)	34 (28.6%)	15 (15.8%)	39 (36.8%)
Do you or your team include others on the unit in your work by weekly group huddles?	Yes	32 (28.1%)	26 (26.3%)	24 (20.2%)	16 (16.8%)	23 (21.7%)
	No	76 (66.7%)	58 (58.6%)	61 (51.3%)	64 (67.4%)	44 (41.5%)
	NA	6 (5.3%)	15 (15.2%)	34 (28.6%)	15 (15.8%)	39 (36.8%)
Do you or your team include others on the unit in your work by other ways?	Yes	20 (17.5%)	14 (14.1%)	16 (13.4%)	14 (14.7%)	10 (9.4%)
	No	87 (76.3%)	70 (70.7%)	69 (58.0%)	66 (69.5%)	57 (53.8%)
	NA	6 (5.3%)	15 (15.2%)	34 (28.6%)	15 (15.8%)	39 (36.8%)
	Missing	1 (0.9%)	—	—	—	—
**Empowerment**						
I am involved in the QI activities in my unit based team	M (SD	3.74 (1.11)	3.36 (1.30)	3.16 (1.51)	3.41 (1.25)	2.85 (1.54)
	NA	6 (5.3%)	15 (15.2%)	34 (28.6)	15 (15.8%)	39 (36.8)
	Missing, N (%)	1 (0.9%)	1 (1.0%)	—	—	—
The person or persons to whom I report most of the time is involved in my QI team's activity	M (SD	3.82 (1.04)	3.52 (1.28)	3.29 (1.55)	3.46 (1.26)	2.86 (1.59)
	NA	6 (5.3%)	15 (15.2%)	34 (28.6)	15 (15.8%)	39 (36.8)
	Missing, N (%)	—	—	1 (0.8%)	1 (1.1%)	—
It is possible for me to make the changes to achieve our quality improvement goals on my unit	M (SD	3.94 (0.81)	3.94 (0.83)	3.80 (0.91)	3.87 (0.69)	4.01 (0.62)
	NA	6 (5.3%)	15 (15.2%)	34 (28.6)	15 (15.8%)	39 (36.8)
	Missing, N (%)	—	—	—	1 (1.1%)	—
I can make the changes needed on my unit to improve resident care	M (SD	3.71 (1.05)	3.46 (1.35)	3 (1.47)	3.38 (1.34)	2.79 (1.48)
	NA	6 (5.3%)	15 (15.2%)	34 (28.6)	15 (15.8%)	39 (36.8)
	Missing, N (%)	—	1 (1.0%)	—	—	—
**Satisfaction with quality of work life**						
Overall I am satisfied with the quality of my work life	M (SD	3.91 (0.88)	4.05 (0.75)	4.04 (0.81)	3.85 (0.97)	3.98 (0.77)
	Missing, N (%)	5 (4.4%)	4 (4.0%)	—	—	1 (0.9%)
