# Supplementary material for: SCOPEOUT: sustainability and spread of quality improvement activities in long-term care- a mixed methods approach
Source: BMC Health Serv Res. 2018 Mar 12;18:174. doi: 10.1186/s12913-018-2978-0 (PMC5848563; doi:10.1186/s12913-018-2978-0)
Supplement: Supplementary file 4 — General Estimating Equation results of the SCOPEOUT survey items. (PDF 853 kb) [file 12913_2018_2978_MOESM4_ESM.pdf]

Quality Improvement Activities

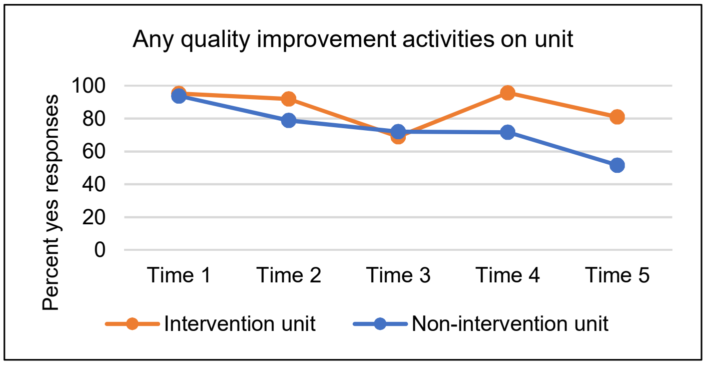

Parameter	OR [95% CI]
Age *(< 25 years = reference)*	
25-34 years	0.764 [0.174; 3.364]
35-44 years	1.119 [0.260; 4.820]
45-54 years	1.222 [0.285; 5.241]
>54 years	0.891 [0.185; 4.285]
Female *(Male = reference)*	1.259 [0.584; 2.712]
Care aide *(Reg. provider= reference)*	0.724 [0.362; 1.447]
Years worked in current role	1.012 [0.970; 1.055]
Years worked on unit	1.010 [0.961; 1.062]
Time	**0.689 [0.591; 0.804]**
Study group *(Non-interv. = reference)*	0.521 [0.157; 1.730]
Time by study group *(Time by non-interv. = reference)*	**1.481 [1.087; 2.019]**
Number of beds	**1.006 [1.002; 1.010]**
Public *(Voluntary = reference)*	2.634 [0.460; 15.088]
SCOPEout rank *(Low = reference)*	
High	**2.365 [1.073; 5.214]**
Medium	**3.505 [1.510; 8.139]**

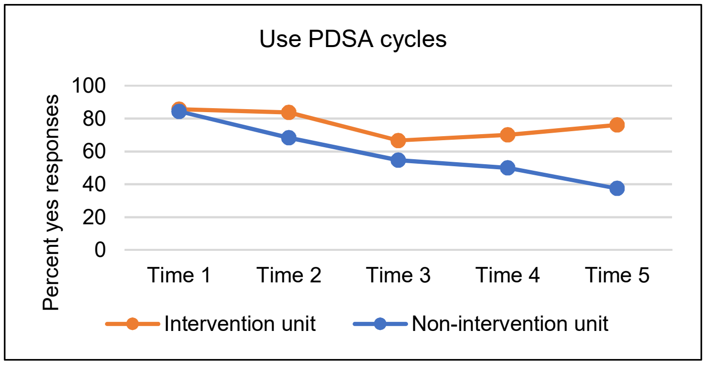

Parameter	OR [95% CI]
Age *(< 25 years = reference)*	
25-34 years	0.571 [0.112; 2.905]
35-44 years	0.951 [0.197; 4.595]
45-54 years	1.060 [0.218; 5.156]
>54 years	0.721 [0.136; 3.812]
Female *(Male = reference)*	1.030 [0.537; 1.977]
Care aide *(Reg. provider= reference)*	1.352 [0.787; 2.325]
Years worked in current role	0.998 [0.963; 1.035]
Years worked on unit	1.021 [0.979; 1.065]
Time	**0.719 [0.620; 0.833]**
Study group *(Non-interv. = reference)*	0.479 [0.163; 1.410]
Time by study group *(Time by non-interv. = reference)*	**1.503 [1.111; 2.034]**
Number of beds	**1.008 [1.005; 1.012]**
Public *(Voluntary = reference)*	**6.529 [1.437; 29.666]**
SCOPEout rank *(Low = reference)*	
High	**5.019 [2.270; 11.097]**
Medium	**4.706 [2.123; 10.432]**

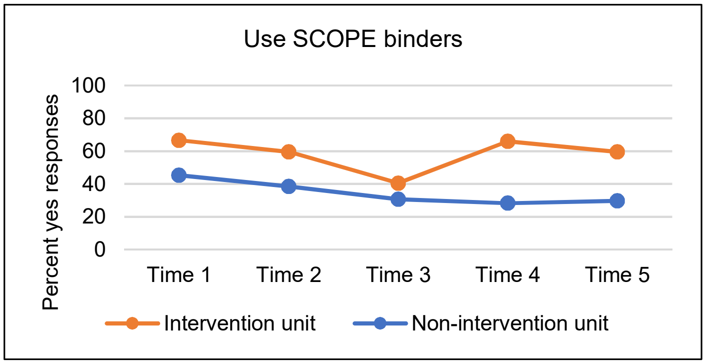

Parameter	OR [95% CI]
Age *(< 25 years = reference)*	
25-34 years	1.810 [0.308; 10.651]
35-44 years	3.267 [0.609; 17.533]
45-54 years	2.370 [0.442; 12.712]
>54 years	1.545 [0.272; 8.759]
Female *(Male = reference)*	1.942 [0.888; 4.247]
Care aide *(Reg. provider= reference)*	1.337 [0.759; 2.356]
Years worked in current role	1.005 [0.973; 1.038]
Years worked on unit	1.036 [0.997; 1.076]
Time	0.946 [0.828; 1.081]
Study group *(Non-interv. = reference)*	1.166 [0.467; 2.910]
Time by study group *(Time by non-interv. = reference)*	1.253 [0.960; 1.637]
Number of beds	**1.011 [1.006; 1.016]**
Public *(Voluntary = reference)*	**30.995 [5.406; 177.710]**
SCOPEout rank *(Low = reference)*	
High	**8.102 [2.939; 22.335]**
Medium	**8.236 [3.119; 21.745]**

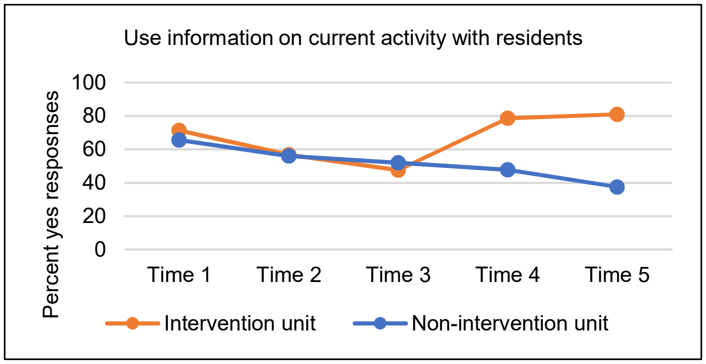

Parameter	OR [95% CI]
Age *(< 25 years = reference)*	
25-34 years	0.339 [0.071; 1.621]
35-44 years	0.548 [0.123; 2.440]
45-54 years	0.470 [0.105; 2.094]
>54 years	0.319 [0.067; 1.514]
Female *(Male = reference)*	**2.180 [1.152; 4.126]**
Care aide *(Reg. provider= reference)*	0.511 [0.300; 0.869]
Years worked in current role	1.005 [0.974; 1.036]
Years worked on unit	1.020 [0.980; 1.060]
Time	0.960 [0.849; 1.086]
Study group *(Non-interv. = reference)*	**0.379 [0.160; 0.897]**
Time by study group *(Time by non-interv. = reference)*	**1.664 [1.296; 2.136]**
Number of beds	**1.004 [1.001; 1.008]**
Public *(Voluntary = reference)*	3.662 [0.865; 15.498]
SCOPEout rank *(Low = reference)*	
High	**2.579 [1.192; 5.582]**
Medium	**3.365 [1.562; 7.249]**

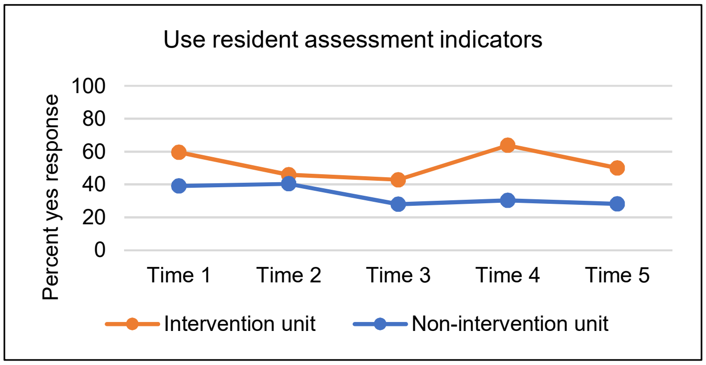

Parameter	OR [95% CI]
Age *(< 25 years = reference)*	
25-34 years	1.406 [0.303; 6.522]
35-44 years	2.294 [0.537; 9.805]
45-54 years	2.014 [0.467; 8.677]
>54 years	1.874 [0.401; 8.767]
Female *(Male = reference)*	1.595 [0.827; 3.076]
Care aide *(Reg. provider= reference)*	0.739 [0.450; 1.216]
Years worked in current role	1.016 [0.987; 1.046]
Years worked on unit	1.010 [0.974; 1.047]
Time	0.955 [0.837; 1.089]
Study group *(Non-interv. = reference)*	0.849 [0.345; 2.085]
Time by study group *(Time by non-interv. = reference)*	**1.309 [1.002; 1.710]**
Number of beds	1.003 [0.999; 1.006]
Public *(Voluntary = reference)*	1.922 [0.452; 8.171]
SCOPEout rank *(Low = reference)*	
High	1.686 [0.756; 3.760]
Medium	**2.415 [1.119; 5.214]**

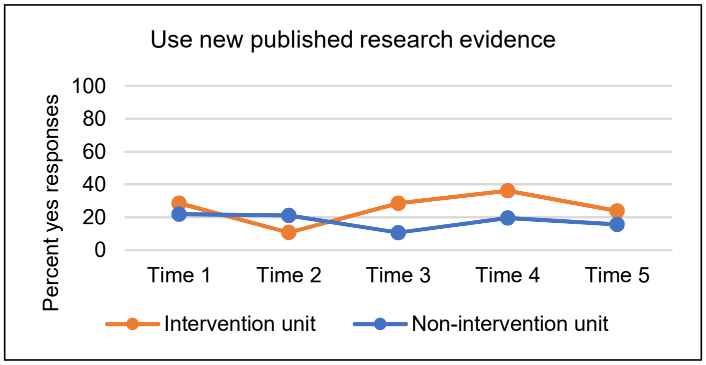

Parameter	OR [95% CI]
Age *(< 25 years = reference)*	
25-34 years	0.462 [0.089; 2.396]
35-44 years	1.006 [0.235; 4.308]
45-54 years	0.397 [0.086; 1.837]
>54 years	0.533 [0.110; 2.571]
Female *(Male = reference)*	2.439 [0.920; 6.463]
Care aide *(Reg. provider= reference)*	1.064 [0.585; 1.935]
Years worked in current role	1.030 [0.995; 1.067]
Years worked on unit	1.014 [0.978; 1.051]
Time	1.010 [0.857; 1.190]
Study group *(Non-interv. = reference)*	0.881 [0.294; 2.637]
Time by study group *(Time by non-interv. = reference)*	1.253 [0.897; 1.750]
Number of beds	**1.003 [1.001; 1.005]**
Public *(Voluntary = reference)*	NA
SCOPEout rank *(Low = reference)*	
High	0.831 [0.376; 1.835]
Medium	1.412 [0.818; 2.438]

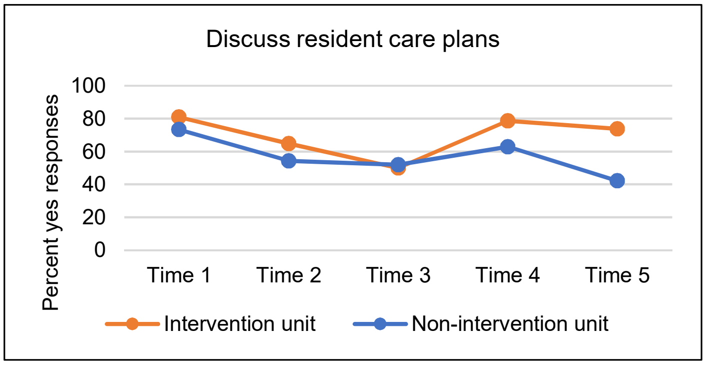

Parameter	OR [95% CI]
Age *(< 25 years = reference)*	
25-34 years	0.286 [0.060; 1.364]
35-44 years	0.358 [0.080; 1.608]
45-54 years	0.382 [0.085; 1.708]
>54 years	0.367 [0.076; 1.781]
Female *(Male = reference)*	1.255 [0.679; 2.317]
Care aide *(Reg. provider= reference)*	0.707 [0.419; 1.192]
Years worked in current role	1.016 [0.983; 1.050]
Years worked on unit	1.013 [0.972; 1.055]
Time	**0.878 [0.774; 0.996]**
Study group *(Non-interv. = reference)*	0.666 [0.271; 1.638]
Time by study group *(Time by non-interv. = reference)*	**1.309 [1.013; 1.691]**
Number of beds	1.003 [0.999; 1.006]
Public *(Voluntary = reference)*	1.333 [0.330; 5.390]
SCOPEout rank *(Low = reference)*	
High	2.002 [0.971; 4.128]
Medium	**2.166 [1.029; 4.562]**

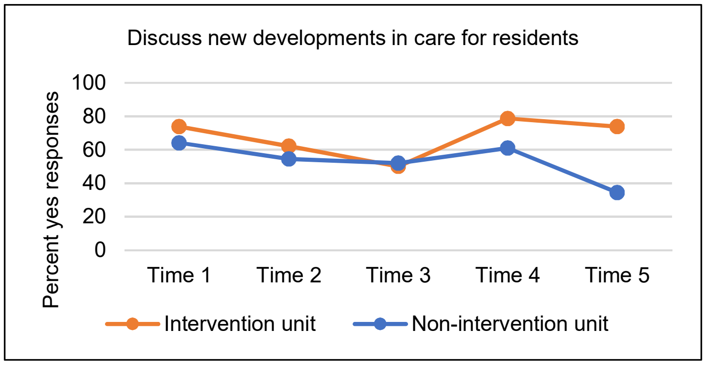

Parameter	OR [95% CI]
Age *(< 25 years = reference)*	
25-34 years	0.468 [0.109; 2.013]
35-44 years	0.598 [0.149; 2.394]
45-54 years	0.707 [0.176; 2.835]
>54 years	0.563 [0.131; 2.413]
Female *(Male = reference)*	1.643 [0.930; 2.901]
Care aide *(Reg. provider= reference)*	0.761 [0.448; 1.292]
Years worked in current role	1.024 [0.993; 1.056]
Years worked on unit	0.996 [0.960; 1.034]
Time	0.914 [0.804; 1.040]
Study group *(Non-interv. = reference)*	0.610 [0.246; 1.516]
Time by study group *(Time by non-interv. = reference)*	**1.372 [1.056; 1.783]**
Number of beds	**1.005 [1.002; 1.009]**
Public *(Voluntary = reference)*	4.086 [0.980; 17.036]
SCOPEout rank *(Low = reference)*	
High	**3.325 [1.622; 6.818]**
Medium	**3.541 [1.702; 7.368]**

Inclusion of Others in Quality Improvement Activities

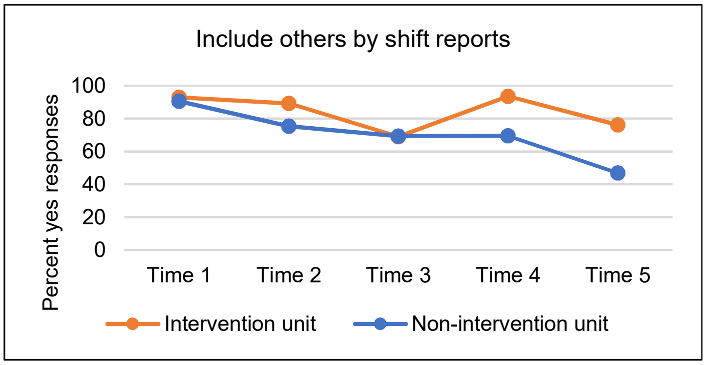

**Parameter**	**OR [95% CI]**
Age *(< 25 years = reference)*	
25-34 years	0.646 [0.145; 2.874]
35-44 years	0.809 [0.187; 3.499]
45-54 years	0.920 [0.213; 3.976]
>54 years	0.659 [0.139; 3.118]
Female *(Male = reference)*	1.313 [0.655; 2.635]
Care aide *(Reg. provider= reference)*	0.845 [0.445; 1.607]
Years worked in current role	1.017 [0.980; 1.057]
Years worked on unit	1.000 [0.958; 1.045]
Time	**0.697 [0.600; 0.810]**
Study group *(Non-interv. = reference)*	0.720 [0.226; 2.292]
Time by study group *(Time by non-interv. = reference)*	**1.357 [1.002; 1.837]**
Number of beds	**1.005 [1.001; 1.009]**
Public *(Voluntary = reference)*	2.247 [0.433; 11.673]
SCOPEout rank *(Low = reference)*	
High	1.923 [0.878; 4.211]
Medium	**3.046 [1.331; 6.973]**

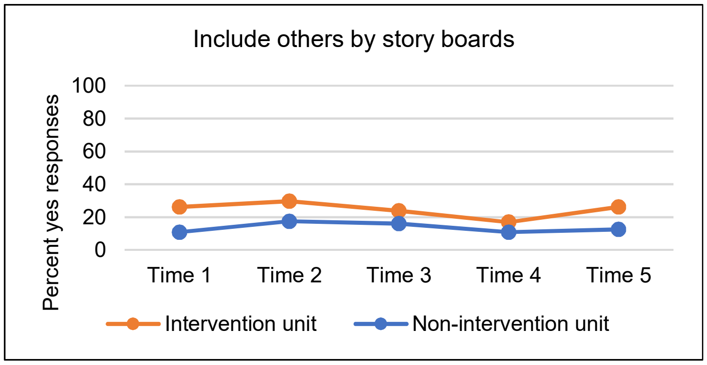

**Parameter**	**OR [95% CI]**
Age *(< 25 years = reference)*	
25-34 years	0.579 [0.095; 3.521]
35-44 years	0.586 [0.109; 3.147]
45-54 years	0.603 [0.113; 3.229]
>54 years	0.277 [0.045; 1.728]
Female *(Male = reference)*	1.300 [0.451; 3.745]
Care aide *(Reg. provider= reference)*	0.817 [0.432; 1.547]
Years worked in current role	0.989 [0.954; 1.025]
Years worked on unit	1.057 [1.013; 1.103]
Time	0.944 [0.806; 1.107]
Study group *(Non-interv. = reference)*	1.917 [0.669; 5.493]
Time by study group *(Time by non-interv. = reference)*	0.979 [0.711; 1.348]
Number of beds	1.007 [1.000; 1.015]
Public *(Voluntary = reference)*	7.147 [0.528; 96.697]
SCOPEout rank *(Low = reference)*	
High	**7.431 [1.557; 35.461]**
Medium	**4.452 [1.049; 18.895]**

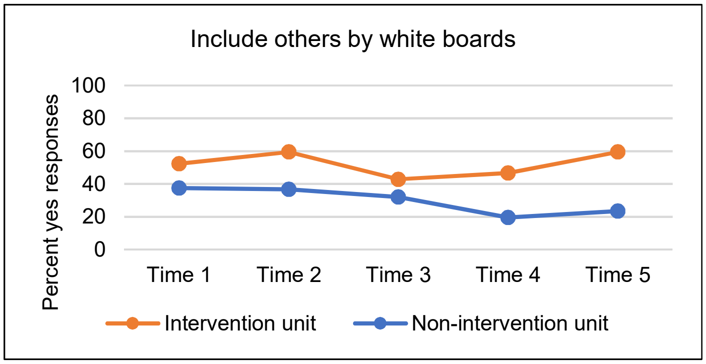

**Parameter**	**OR [95% CI]**
Age *(< 25 years = reference)*	
25-34 years	0.859 [0.170; 4.346]
35-44 years	0.571 [0.124; 2.624]
45-54 years	0.829 [0.182; 3.786]
>54 years	0.591 [0.120; 2.907]
Female *(Male = reference)*	1.224 [0.638; 2.349]
Care aide *(Reg. provider= reference)*	0.690 [0.416; 1.146]
Years worked in current role	1.025 [0.997; 1.055]
Years worked on unit	1.017 [0.982; 1.053]
Time	0.931 [0.815; 1.062]
Study group *(Non-interv. = reference)*	0.986 [0.408; 2.384]
Time by study group *(Time by non-interv. = reference)*	**1.301 [1.003; 1.689]**
Number of beds	1.004 [0.999; 1.009]
Public *(Voluntary = reference)*	3.087 [0.531; 17.949]
SCOPEout rank *(Low = reference)*	
High	**5.030 [1.890; 13.391]**
Medium	**5.499 [2.164; 13.976]**

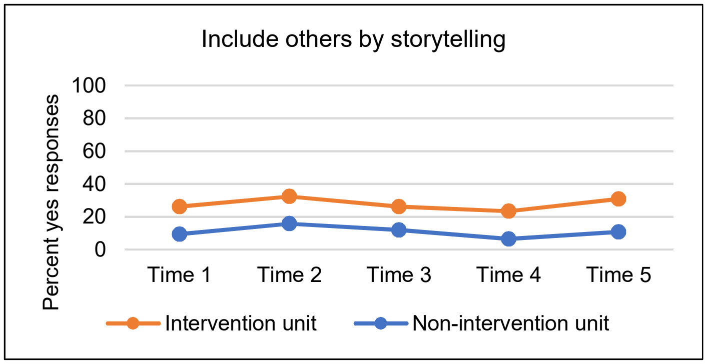

**Parameter**	**OR [95% CI]**
Age *(< 25 years = reference)*	
25-34 years	0.194 [0.033; 1.136]
35-44 years	0.171 [0.032; 0.914]
45-54 years	0.300 [0.057; 1.561]
>54 years	0.239 [0.040; 1.413]
Female *(Male = reference)*	1.287 [0.431; 3.843]
Care aide *(Reg. provider= reference)*	1.124 [0.573; 2.205]
Years worked in current role	0.991 [0.957; 1.027]
Years worked on unit	1.015 [0.972; 1.059]
Time	1.032 [0.871; 1.222]
Study group *(Non-interv. = reference)*	2.839 [0.861; 9.364]
Time by study group *(Time by non-interv. = reference)*	0.966 [0.679; 1.375]
Number of beds	1.006 [0.999; 1.014]
Public *(Voluntary = reference)*	4.014 [0.270; 59.737]
SCOPEout rank *(Low = reference)*	
High	3.879 [0.765; 19.660]
Medium	**5.730 [1.326; 24.767]**

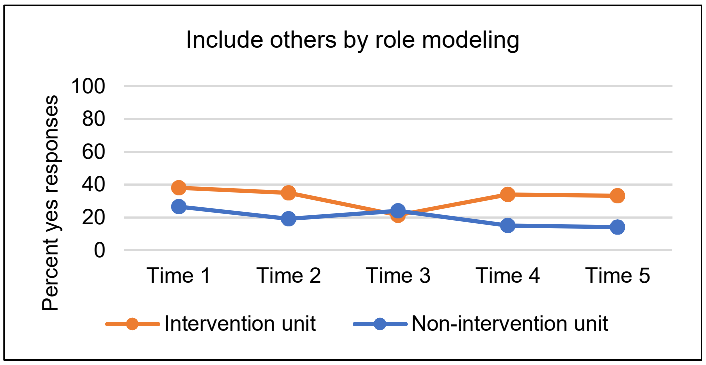

Parameter	OR [95% CI]
Age *(< 25 years = reference)*	
25-34 years	0.390 [0.056; 2.727]
35-44 years	1.300 [0.257; 6.592]
45-54 years	1.655 [0.327; 8.385]
>54 years	1.412 [0.260; 7.680]
Female *(Male = reference)*	1.476 [0.641; 3.402]
Care aide *(Reg. provider= reference)*	**0.426 [0.247; 0.732]**
Years worked in current role	0.999 [0.969; 1.029]
Years worked on unit	0.996 [0.959; 1.035]
Time	0.950 [0.833; 1.082]
Study group *(Non-interv. = reference)*	1.631 [0.644; 4.133]
Time by study group *(Time by non-interv. = reference)*	1.059 [0.817; 1.373]
Number of beds	**1.006 [1.001; 1.011]**
Public *(Voluntary = reference)*	4.161 [0.533; 32.459]
SCOPEout rank *(Low = reference)*	
High	**3.540 [1.046; 11.983]**
Medium	2.190 [0.678; 7.075]

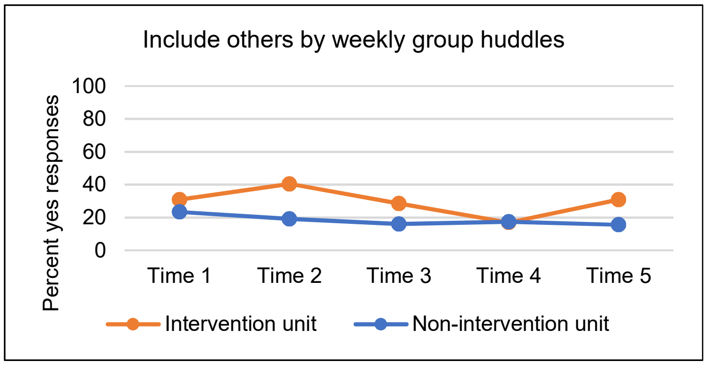

Parameter	OR [95% CI]
Age *(< 25 years = reference)*	
25-34 years	1.974 [0.229; 17.010]
35-44 years	1.844 [0.239; 14.214]
45-54 years	1.734 [0.223; 13.454]
>54 years	1.917 [0.229; 16.028]
Female *(Male = reference)*	1.621 [0.677; 3.884]
Care aide *(Reg. provider= reference)*	**0.458 [0.266; 0.790]**
Years worked in current role	1.018 [0.983; 1.055]
Years worked on unit	1.019 [0.979; 1.060]
Time	0.884 [0.753; 1.037]
Study group *(Non-interv. = reference)*	1.606 [0.538; 4.800]
Time by study group *(Time by non-interv. = reference)*	1.034 [0.749; 1.426]
Number of beds	**1.007 [1.001; 1.013]**
Public *(Voluntary = reference)*	3.782 [0.424; 33.714]
SCOPEout rank *(Low = reference)*	
High	**8.388 [2.404; 29.273]**
Medium	2.547 [0.743; 8.732]

Empowerment and Satisfaction with Quality of Worklife

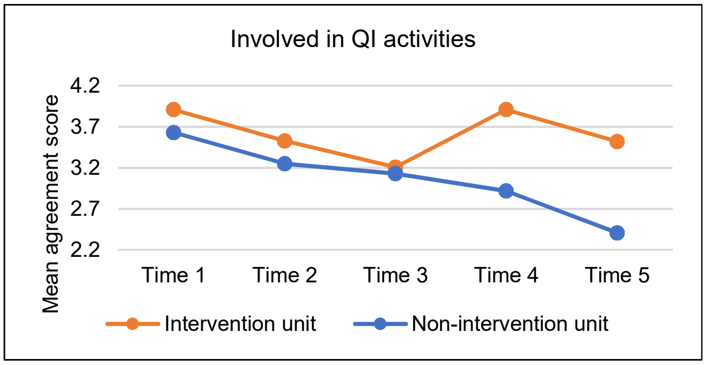

Parameter	OR [95% CI]
Age *(< 25 years = reference)*	
25-34 years	0.064 [-0.772; 0.900]
35-44 years	0.351 [-0.435; 1.137]
45-54 years	0.341 [-0.444; 1.126]
>54 years	0.192 [-0.648; 1.033]
Female *(Male = reference)*	0.170 [-0.199; 0.540]
Care aide *(Reg. provider= reference)*	-0.247 [-0.537; 0.042]
Years worked in current role	0.00 [-0.018; 0.018]
Years worked on unit	**0.025 [0.003; 0.047]**
Time	**-0.276 [-0.373; -0.179]**
Study group *(Non-interv. = reference)*	-0.400 [-0.847; 0.048]
Time by study group *(Time by non-interv. = reference)*	**0.271 [0.138; 0.405]**
Alberta *(British Columbia = reference)*	**6.115 [2.035; 10.195]**
Number of beds	**-0.024 [-0.043; -0.005]**
Public *(Voluntary = reference)*	**-2.726 [-5.449; -0.004]**
SCOPEout rank *(Low = reference)*	
High	**0.468 [0.023; 0.914]**
Medium	**1.075 [0.616; 1.535]**

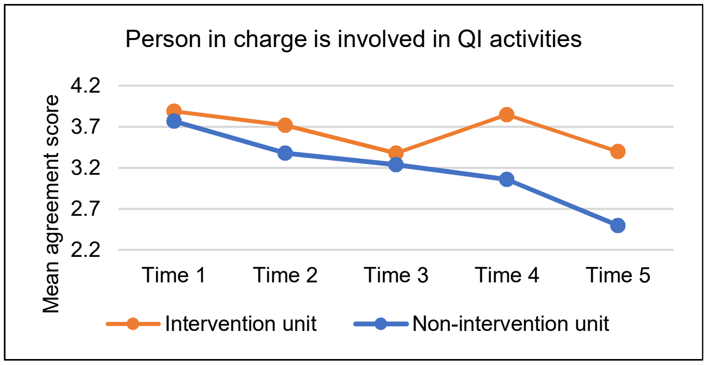

Parameter	OR [95% CI]
Age *(< 25 years = reference)*	
25-34 years	-0.357 [-1.139; 0.425]
35-44 years	-0.223 [-0.955; 0.510]
45-54 years	-0.250 [-0.970; 0.470]
>54 years	-0.477 [-1.250; 0.297]
Female *(Male = reference)*	0.131 [-0.239; 0.500]
Care aide *(Reg. provider= reference)*	**-0.375 [-0.698; -0.052]**
Years worked in current role	0.009 [-0.010; 0.028]
Years worked on unit	0.016 [-0.007; 0.038]
Time	-0.273 [-0.375; -0.170]
Study group *(Non-interv. = reference)*	-0.305 [-0.760; 0.150]
Time by study group *(Time by non-interv. = reference)*	**0.209 [0.066; 0.351]**
Alberta *(British Columbia = reference)*	**6.195 [2.539; 9.852]**
Number of beds	**-0.025 [-0.041; -0.008]**
Public *(Voluntary = reference)*	**-2.989 [-5.501; -0.477]**
SCOPEout rank *(Low = reference)*	
High	0.409 [-0.053; 0.871]
Medium	**1.097 [0.642; 1.553]**

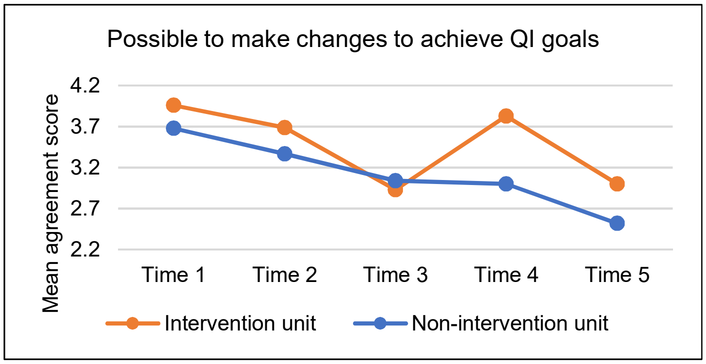

Parameter	OR [95% CI]
Age *(< 25 years = reference)*	
25-34 years	-0.208 [-0.923; 0.508]
35-44 years	-0.020 [-0.688; 0.649]
45-54 years	-0.127 [-0.785; 0.530]
>54 years	-0.189 [-0.904; 0.526]
Female *(Male = reference)*	0.045 [-0.345; 0.435]
Care aide *(Reg. provider= reference)*	-0.268 [-0.570; 0.033]
Years worked in current role	0.006 [-0.011; 0.024]
Years worked on unit	0.021 [-0.001; 0.043]
Time	**-0.278 [-0.377; -0.180]**
Study group *(Non-interv. = reference)*	-0.386 [-0.836; 0.064]
Time by study group *(Time by non-interv. = reference)*	**0.240 [0.102; 0.379]**
Alberta *(British Columbia = reference)*	**5.120 [1.289; 8.951]**
Number of beds	**-0.020 [-0.037; -0.002]**
Public *(Voluntary = reference)*	-2.033 [-4.635; 0.569]
SCOPEout rank *(Low = reference)*	
High	**0.558 [0.095; 1.021]**
Medium	**1.074 [0.619; 1.529]**

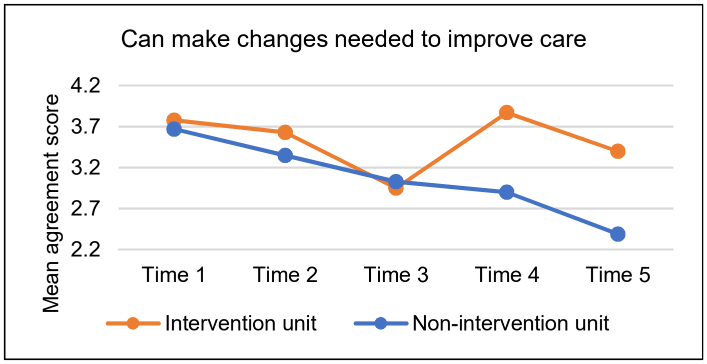

Parameter	OR [95% CI]
Age *(< 25 years = reference)*	
25-34 years	-0.079 [-0.807; 0.650]
35-44 years	-0.058 [-0.727; 0.612]
45-54 years	-0.028 [-0.694; 0.638]
>54 years	-0.241 [-0.968; 0.485]
Female *(Male = reference)*	0.014 [-0.407; 0.434]
Care aide *(Reg. provider= reference)*	**-0.377 [-0.686; -0.069]**
Years worked in current role	0.002 [-0.017; 0.021]
Years worked on unit	0.022 [-0.001; 0.045]
Time	**-0.304 [-0.401; -0.208]**
Study group *(Non-interv. = reference)*	-0.453 [-0.911; 0.006]
Time by study group *(Time by non-interv. = reference)*	**0.275 [0.138; 0.412]**
Alberta *(British Columbia = reference)*	**4.308 [0.457; 8.159]**
Number of beds	-0.016 [-0.034; 0.001]
Public *(Voluntary = reference)*	-1.703 [-4.308; 0.902]
SCOPEout rank *(Low = reference)*	
High	0.458 [-0.018; 0.934]
Medium	**0.876 [0.403; 1.348]**
